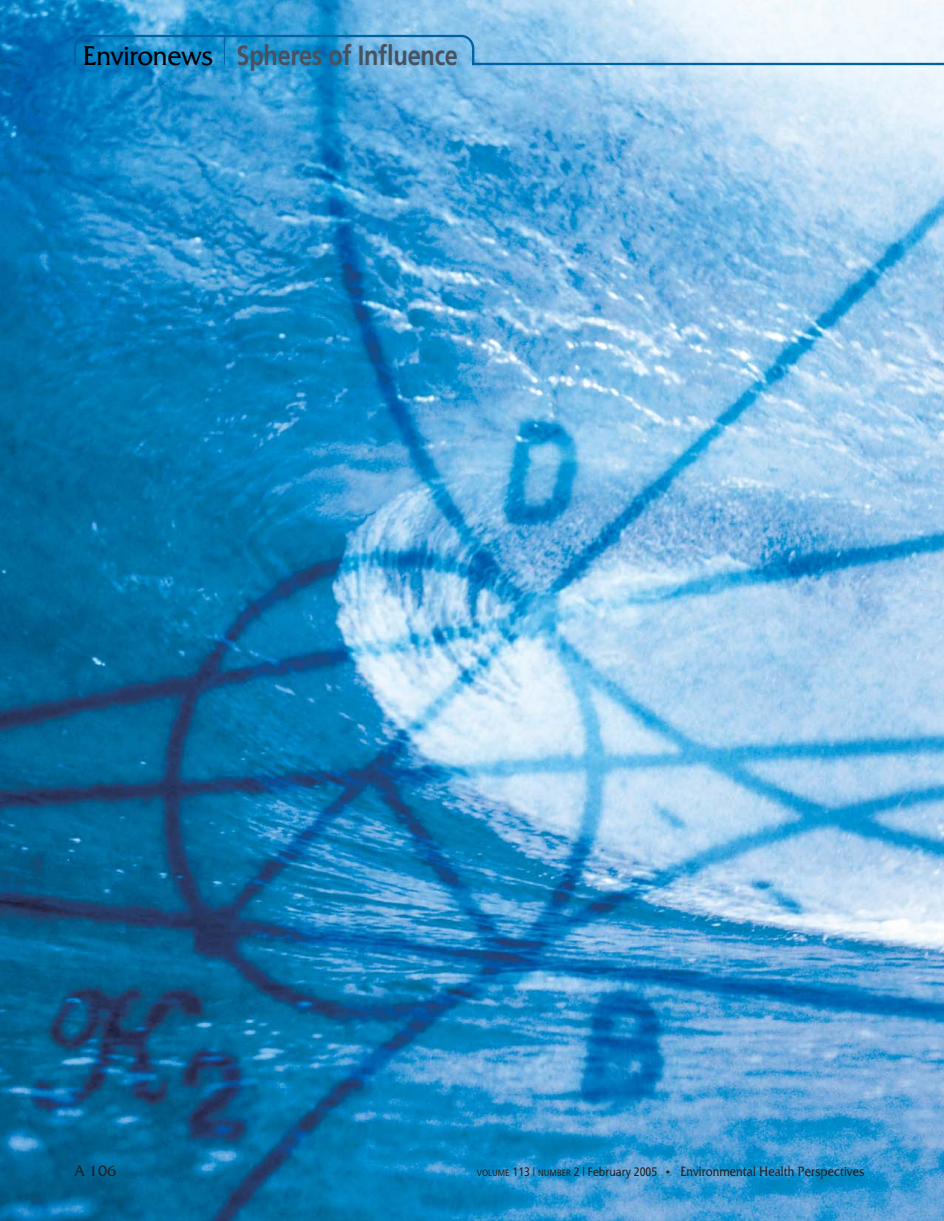# America’s Oceans: A Blueprint for the Future

**DOI:** 10.1289/ehp.113-a106

**Published:** 2005-02

**Authors:** John Tibbetts

The nation’s coasts and oceans are undervalued as an economic force, their ecological value is often misunderstood, and as a resource they are often poorly managed. Around the nation, coastal communities are growing at an explosive pace, but the federal government has struggled to respond, shackled by a fractured system of responsibility and authority over coastal and marine ecosystems. On many key issues in federal ocean policy, there has been a sense of partial paralysis. That’s according to the U.S. Commission on Ocean Policy’s final report released on 20 September 2004. Calling for measures to repair the nation’s ocean governance system, the commission sent its report, *An Ocean Blueprint for the 21st Century*, to President Bush and Congress. Responding to the commission, President Bush signed an executive order in December 2004 to create the Committee on Ocean Policy, which will oversee the nation’s ocean and coastal management.

The report offers the most comprehensive national assessment of U.S. oceans in 35 years, since the 1969 report of the Stratton Commission. That commission was created by Congress to provide a comprehensive assessment of U.S. ocean policy. Since that earlier report was issued, however, the federal government has taken what the latest report calls an “ad hoc approach” to ocean and coastal policy.

In the 1970s, new federal laws including the Magnuson-Stevens Fishery Conservation and Management Act and the Coastal Zone Management Act (CZMA) addressed urgent needs, but, says the report, they lacked an overarching vision critical to a coherent national ocean policy. Today, more than 55 congressional committees and subcommittees oversee at least 20 federal agencies and permanent commissions that implement more than 140 federal ocean-related laws.

Federal agencies usually regulate separately each industrial sector in marine and coastal zones—if sectors are regulated at all. Local and state agencies, moreover, manage resources according to traditional political boundaries, not according to ecosystem boundaries such as watersheds. But ecosystems don’t stop at political boundaries, and mismanagement in one area can affect many other areas, too. Dozens of agencies and jurisdictions, often working separately, are responsible for managing land uses and impacts in coastal areas and related offshore marine areas.

In 2001, President Bush appointed the U.S. Commission on Ocean Policy—a mix of 16 academics, business executives, and naval officers—to make recommendations on how to improve the capacity of the nation to manage ocean- and coast-related activities. On 20 April 2004, the commission released a preliminary report to state governors and the public for comment.

Once all comments were considered, the commission delivered the final report with 212 recommendations and formally disbanded, having discharged its duty. Its findings are grim—the commissioners write that the failure to properly manage the nation’s coasts and oceans is “compromising [these resources’] ecological integrity, diminishing our ability to fully realize their potential, costing us jobs and revenue, threatening human health, and putting our future at risk.”

## Coastal Chaos

The nation’s oceans, coasts, and Great Lakes are greatly important to American prosperity, the commissioners write. Based on year 2000 estimates, ocean-related activities directly contributed more than $117 billion to the nation’s economy and supported more than 2 million jobs. Every year, hundreds of millions of people visit U.S. coastlines; tourism and recreation is the fastest-growing job sector in coastal counties nationally.

Coastal states accounted for more than three-quarters of the U.S. economy in 2000, measured by gross domestic product, according to Charles Colgan, a University of Southern Maine economist who, with his colleagues, is conducting the ongoing National Ocean Economics Program, the first comprehensive study of economic and social changes along U.S. coastlines. Coastal-zone counties, moreover, accounted for one-third of the nation’s gross domestic product in 2000.

The CZMA, passed initially in 1972, provides federal funds to states, which in turn manage their coastal areas in accordance with a set of federal guidelines. Each state’s coastal zone management program is unique, but many are primarily permitting programs for land-based development along near-shore areas.

Coastal zone management in many states addresses a relatively narrow strip of land along the shoreline. Yet the coastal economy includes metropolitan areas and watersheds that spread many miles inland. From 1990 to 2000, U.S. near-coast areas—those zip codes closest to the shoreline—had 35% job growth but only 11% population growth, says Colgan.

Greater numbers of people are working in industries located near the shoreline, but they increasingly live in rapidly expanding inland suburbs—partly because jobs in the recreation and tourism sector tend to be relatively low-wage. “Because people are moving farther and farther away from the near-coast, we now have a jobs–housing mismatch,” says Colgan. “We are changing the nature of the ecosystem, opening huge inland areas for development.”

## A New Maxim for Management

The success of U.S. coastal communities has had environmental costs. Currently, when a project—whether it be a golf course or a fish farm—is proposed along the coast or in the sea, it is considered for permitting in almost an ecological vacuum, as if no other projects were taking place that alter wetlands or the beach or marine habitats.

Today’s spread-out development destroys forests and paves over farmland for many miles from the coast. Rainfall and snowmelt wash pollutants such as pesticides, fertilizers, motor oil, bacteria, viruses, pet waste, chemicals, sediments, and other non–point source pollutants off lawns, roads, and parking lots into waterways that flow to the ocean.

Non–point source pollutants are the primary cause of water quality problems in U.S. estuaries, greatly contributing to nutrient enrichment, oxygen depletion of surface waters, harmful algal blooms, and toxic contaminants, according to the report. Excess nutrients and pollution released into the ocean, combined with a rise in ocean surface temperatures, are causing an increase in pathogens (primarily bacteria and viruses) in marine waters. These same environmental conditions can also promote algal blooms, some of which produce toxins that can harm human health, with effects ranging from irritating to deadly.

To help combat these threats, the commission’s report recommends a new direction of “ecosystem-based management” and regional decision making. This means taking into account ecological relationships between coastal watersheds and coastal oceans, and allowing management to cross government’s jurisdictional lines.

“We tried to get a management construct in mind that would push us all to think about what’s happening in watersheds and what ultimately happens in coastal ocean waters and resources,” says commissioner Paul Sandifer, a senior scientist for the National Oceanic and Atmospheric Administration (NOAA) National Centers for Coastal Ocean Science. “We recommend that watersheds need to be connected to coastal and ocean systems in regional planning activities, and this includes taking into account increasing urbanization and suburbanization.”

“Ecosystem-based management is extremely important for fisheries,” says commissioner Andrew Rosenberg, a fisheries scientist at the University of New Hampshire. “Managing fisheries in isolation from other sectors doesn’t make a lot of sense.” Adds Christopher Mann, policy director for the now-closed nonprofit Center for SeaChange: “We’ve treated the ocean largely like a fish bowl without habitat but with a bunch of fish in it. We need to realize that fish are produced by the ecosystem.”

The commission’s report calls for major changes to CZMA programs, including reauthorizing the act to enable states to incorporate a coastal watershed focus. Congress, moreover, should require CZMA programs to do more research on coastal and marine ecosystems, assess resources, and create measurable water pollution reduction goals, especially for non–point source pollution.

## Plumbing the Ocean Depths

The report urges the federal government to double the budget for ocean research (now $650 million annually) over the next five years. “We are currently underinvesting in our oceans,” says David Festa, director of the Oceans Program at Environmental Defense, a nonprofit organization. “The full impact of the benefits that are possible from a revitalized approach to our oceans depends on adequate funding.”

The report calls on NOAA, the National Science Foundation (NSF), and the NIEHS to support expanded research in marine microbiology and virology. Agencies should also support the development of better methods to monitor and identify pathogens and chemical toxicants in ocean and coastal waters and organisms.

Congress, the commissioners write, should establish a national, multiagency Oceans and Human Health Initiative to coordinate and sponsor exploration, research, and new technologies that would address connections among the oceans, ecosystems, and human health. NOAA’s current Oceans and Human Health Initiative and the NIEHS–NSF Centers for Oceans and Human Health should be expanded and coordinated as the basis for this initiative, according to the report. The NOAA Oceans and Human Health Initiative coordinates agency activities and focuses funding on ocean and health issues including infectious diseases, harmful algal blooms, environmental indicators, climate, and marine biomedicine. The joint Centers for Oceans and Human Health promote interdisciplinary collaborations among biomedical and ocean scientists, with the goal of improving knowledge about the impacts of the ocean on human health.

The marine environment is the greatest source of biological diversity on the planet. By collecting specimens in places like coral reefs that have extremely high biodiversity, scientists are more likely to find compounds that could be used to make novel drugs. The report calls for NOAA, the NSF, and the NIEHS to expand efforts to study the evolution, ecology, chemistry, and molecular biology of marine species, discover potential marine products, and develop practical compounds for new medicines.

Moreover, NOAA, the Environmental Protection Agency, and the Food and Drug Administration (FDA), working with state and local governments, should fully implement existing programs to protect human health from contaminated seafood and coastal waters. NOAA monitors fisheries management through the Seafood Inspection Program. At the same time, the FDA is responsible for ensuring the safety of imported and domestic seafood sold in the United States. The FDA’s Hazard Analysis and Critical Control Point system, implemented in 1997, requires both U.S. producers and foreign importers to analyze potential hazards in preparing, handling, and packaging seafood and to implement plans to control hazards. Meanwhile, states, territories, and tribes issue fish and wildlife consumption advisories, which are based on Environmental Protection Agency guidance. These advisories include recommendations about limiting consumption of certain fish and seafood harvested from particular water bodies.

The commission report calls for better seafood screening, processing regulations, ocean monitoring, and public advisories. The nation needs more rapid, accurate, and cost-effective techniques for detecting pathogens and toxins in seafood. New techniques should be incorporated into seafood safety and surveillance programs, especially inspections of imported seafood and aquaculture products.

## Finding a Focus for Policy

The nation needs a focused ocean policy, according to the commission. The report calls for strengthening NOAA—which currently lacks public visibility and budget support—as the lead oceans agency in the federal government. The report says that Congress should pass a law ensuring that NOAA’s structure is consistent with the principles of ecosystem-based management. The report also calls for a strengthened science program and a more service-oriented approach, with budget support to address its responsibilities. Further, the president should create a National Ocean Council within the White House to develop and direct ocean policy.

According to the commissioners, a national council is needed because of the rapidly growing new uses of marine and coastal resources, including fish farming, renewable ocean power, desalination plants for drinking water, deep-sea mining, and bio-prospecting for new drugs. The federal government lacks a mechanism to address these new uses in a comprehensive way, says commissioner Marc J. Hershman, an ocean policy professor at the University of Washington. “Here we have the domain of the ocean over which [the government has] virtually full responsibility, and we’re only managing existing uses out there. The country has got to look seaward, and one way to do that is to create a mechanism for licensing new activities and doing test cases and thinking about these resources long-term.”

As envisioned by the commission, this council would be a permanent cabinet-level group providing high-level attention to ocean issues and helping to ensure that all agencies comply with national ocean policy and standards. The National Ocean Council would also provide a point of contact for management of ocean- and coast-related activities. The proposed council, working with states, should make reduction of non–point source pollution in coastal watersheds a national goal, according to the report.

The commission also recommends that Congress create an Ocean Policy Trust Fund, which would draw revenue from offshore oil and gas development and “other offshore uses” (for example fish farming, deep-sea mining, and bioprospecting for new drugs) to pay for the eventual $3.2 billion annual cost of implementing the report recommendations.

The report endorses voluntary regional councils comprising state, federal, local, and tribal leaders. Each region would determine what its own most important problems are, and work to find solutions. The commission also urges the creation of a comprehensive national network of ocean observatories that would help better track changes in marine conditions such as toxic algal blooms.

Also at the regional level, the commission calls for significant changes in fishery management councils, and encourages market-based individual fishing quotas that would allow commercial fishermen to buy and trade their “dedicated-access” privileges. Many U.S. commercial fish stocks have collapsed due to overexploitation. When important fish stocks have declined rapidly, some regional fishery councils have not significantly reduced fishermen’s access to those fisheries long enough to bring them back to health, according to Rosenberg.

The commission recommends that regional fishery councils rely exclusively on scientific advice to determine how many fish in specific stocks can be caught without further depleting those stocks. “Councils would be bound to set conservation limits on the basis of scientific advice, and they would have to stay within those limits,” says Rosenberg. In the past, economic considerations—for example, estimates of job losses in fishing industries—have sometimes trumped scientific advice on the sustainable harvests of fishing stocks. However, over the past decade, most regional councils have made significant progress toward following scientific advice on conservation limits, Rosenberg says.

## The White House Response

In December 2004, Congress passed the Oceans and Human Health Act, which the president signed into the law. The law authorizes a coordinated national research program to improve understanding of the role of oceans in human health.

In response to the report, President Bush on 17 December 2004 issued an executive order creating a cabinet-level Committee on Ocean Policy that would consider ways to better manage oceans and coastlines. James L. Connaughton, chairman of the White House Council on Environmental Quality, will chair the committee.

In its new U.S. Ocean Action Plan, the White House is acting immediately on 40 of the commission’s 212 recommendations. Among other initiatives, the administration will work with regional fishery councils to promote greater use of individual fishing quotas, work toward building a global Earth observation network, including integrated ocean observation, and develop an ocean research priorities plan and implementation strategy. The White House plan does not address the commission’s major proposed changes such as creating a trust fund for new ocean initiatives. The only new funding announced was $2.7 million that will be requested in the fiscal year 2006 budget for coral reef improvements.

However, there is consensus in Washington that the nation’s ocean policy system is indeed fractured and in need of reform. “The White House acknowledges the problem: that the diagnosis by the commission is accurate, that we need change,” says Festa. He expects the White House to continue pursuing discrete changes, which the administration is willing to implement quickly, and then develop a process to tackle some of the hard problems. The administration, he says, is adopting the philosophy that big change is achieved in incremental steps, “but the status quo has a lot of inertia.”

## Figures and Tables

**Figure:**